# Bridging Scales: Mesoscience as a Transformative Methodology

**DOI:** 10.1002/cai2.70033

**Published:** 2025-11-09

**Authors:** Raffaella Ocone

**Affiliations:** ^1^ Institute of GeoEnergy Engineering Heriot‐Watt University Edinburgh UK

The paper “Challenges in Immune System: Mesoscale and mesoregime complexity” by Ren et al. [1] represents more than an important advance in immunology; it offers a growing recognition of mesoscience as a unifying scientific framework for understanding complexity. By applying the mesoscience approach to the immune system, the authors demonstrate the power and versatility of a methodology first developed by Prof. Li and colleagues and refined over more than three decades of research [2]. Grounded in the principle of *“compromise in competition”*, mesoscience focuses on the mesoscale, the critical scale between microscopic and macroscopic scales, where highly relevant phenomena happen. Mesoscale has already proven its high relevance in engineering. This new contribution exemplifies its equally profound potential in the life sciences and indeed in other scientific domains.

For decades, immunology has thrived on the molecular and cellular levels, revealing fundamental mechanisms underlying receptor–ligand interactions, signal transduction, gene regulation and cell differentiation. Traditionally, in studying and modelling the immune system, a reductionist approach has been followed; such an approach, whilst foundational, captures only a fraction of the immune system's complexity. Immunity is not simply a molecular phenomenon, but it is a dynamic, multilevel system essential for maintaining organismal homoeostasis.

The immune system integrates both innate and adaptive components, offering broad‐spectrum and antigen‐specific defences, respectively. Beyond pathogen clearance, it contributes to tissue remodelling, wound healing, tumour surveillance and microbiota regulation, illustrating its operation across biomolecular, cellular, tissue, and organismal levels. At the heart of this complexity are mesoscale structures, namely spatiotemporally dynamic organisational frameworks that link local molecular events to global immune behaviours. These mesoscale structures are “nested” across all four levels of organisation (i.e., biomolecular, cellular, organ and organismal levels), making a meso‐science perspective essential to fully reveal the system's complexity. Most immunological research remains confined to molecular and cellular layers. Omics technologies, while powerful, often produce fragmented snapshots of a system in motion. The mesoscale, by contrast, offers a conceptual and analytical bridge, one that can connect the molecular detail to the system‐level function.

The immune system operates through *nested mesoscale structures*, spanning biomolecular, cellular, tissue and organismal levels. These structures govern how local interactions propagate upward, influencing processes as diverse as tissue remodelling, tumour surveillance, and host–microbiota balance.

The paper by Ren and coauthors provides an extremely timely illustration of the high potential of mesoscience in immunology. Based on the integration of techniques readily available, such as high‐resolution imaging, spatial proteomics and single‐cell approaches with multiscale computational modelling, it is shown that the mesoscience framework can capture how local molecular processes generate mesoscale patterns that shape immune behaviour. This not only offers new ways to interrogate the immune system but also opens a path towards predictive modelling, which is proposed as an essential step for advancing next‐generation therapies. Importantly, the work also underscores the limits of reductionism. Many of the immune system's most pressing questions, from drug delivery barriers to microenvironmental resistance, are mesoscale in nature and cannot be resolved by molecular analysis alone.

The significance of this contribution, however, extends far beyond immunology. Mesoscience provides a rigorous, unifying framework for addressing complexity across disciplines, from engineering and physics to biology, environmental science and medicine. By focusing on the intermediate scale where competing forces interact and compromise, mesoscience enables researchers to reveal the organising principles that govern complex systems. In doing so, it offers not merely new tools but a new way of thinking.

This is a call to action. The integration of mesoscience into more areas of enquiry has the potential to reshape the scientific landscape. Realising this vision will require more than disciplinary excellence; it demands active, sustained collaboration among fields, involving molecular biologists, engineers, physicists, clinicians, data scientists and experimentalists. High‐quality collaborations, together with the sharing of data, will be essential to converge towards a scientific framework that will solve problems and explain mesoscale patterns and phenomena.

The paper exemplifies how mesoscience can move a field forward. But it also points to something larger: the emergence of a new scientific language for complexity, one capable of transcending traditional boundaries. Embracing this framework will not only advance immunology but also provide a powerful platform for discovery across the sciences.

## Author Contributions

**Raffaella Ocone:** writing and proofing.

## Ethics Statement

The author has nothing to report.

## Consent

The author has nothing to report.

## Conflicts of Interest

The author declares no conflicts of interest.

## Data Availability

The author has nothing to report.
